# Zoledronate Sequential Therapy After Denosumab Discontinuation to Prevent Bone Mineral Density Reduction: A Randomized Clinical Trial

**DOI:** 10.1001/jamanetworkopen.2024.43899

**Published:** 2024-11-11

**Authors:** Chia-Che Lee, Chen-Yu Wang, Hung-Kuan Yen, Chih-Chien Hung, Cheng-Yo Lai, Ming-Hsiao Hu, Ting-Ming Wang, Chung-Yi Li, Shau-Huai Fu

**Affiliations:** 1 Department of Orthopedic Surgery, National Taiwan University Hospital, Taipei, Taiwan; 2 Graduate Institute of Biomedical Electronics and Bioinformatics, National Taiwan University, Taipei, Taiwan; 3 National Center for Geriatrics and Welfare Research, National Health Research Institutes, Yunlin, Taiwan; 4 Department of Pharmacy, National Taiwan University Hospital Yunlin Branch, Douliu, Taiwan; 5 Department of Orthopedics, National Taiwan University Hospital Yunlin Branch, Douliu, Taiwan; 6 Department of Orthopaedic Surgery, National Taiwan University Hospital, Hsinchu, Taiwan; 7 Department of Public Health, College of Medicine, National Cheng Kung University, Tainan, Taiwan; 8 Department of Public Health, College of Public Health, China Medical University, Taichung, Taiwan; 9 Department of Healthcare Administration, College of Medical and Health Science, Asia University, Taichung, Taiwan

## Abstract

**Importance:**

Discontinuation of denosumab without transitioning to another antiresorptive agent results in rapid bone loss and an increased risk of fracture. Previous randomized studies reported inconsistent results regarding the efficacy of zoledronate as sequential therapy.

**Objective:**

To investigate the use of sequential therapy with zoledronate to prevent bone loss and decreased bone mineral density (BMD) after denosumab discontinuation in the first year.

**Design, Setting, and Participants:**

The Denosumab Sequential Therapy prospective, open-label, parallel-group randomized clinical trial was conducted at a referral center and 2 affiliated hospitals in Taiwan. Recruitment was conducted from April 1, 2019, to May 31, 2021, and a 2-year follow-up was planned. The trial included postmenopausal women and men aged 50 years or older who received regular denosumab treatment for at least 2 years and did not have previous exposure to other antiosteoporosis medication or meet other exclusion criteria.

**Intervention:**

Participants were assigned via stratified randomization to 1 of 2 groups: group A received continuous denosumab treatment (60 mg twice yearly) as the positive control, whereas group ZOL received 1 dose of zoledronate (5 mg) in the first year.

**Main Outcomes and Measures:**

The coprimary outcomes were BMD percentage changes in the lumbar spine (LS-BMD), total hip (TH-BMD), and femoral neck (FN-BMD), respectively. An intention-to-treat analysis was performed.

**Results:**

This study included 101 patients (95 women [94.1%]; median age, 72.0 [IQR, 67.0-76.0] years). There were 25 patients in group A (23 women [92.0%]; median age, 74.0 [IQR, 70.0 to 78.0] years) and 76 in group ZOL (72 women [94.7%]; median age, 71.0 [IQR, 65.7 to 76.0] years). In the first year, group ZOL had a significant median decrease in LS-BMD (−0.68% [IQR, −3.22% to 2.75%]) compared with group A (1.30% [IQR, −0.68% to 5.24%]) (*P* = .03). No significant differences between groups A and ZOL were observed for TH-BMD (median, 1.12% [IQR, −0.06% to 2.25%] vs 0% [−1.47% to 2.15%]) (*P* = .24) and FN-BMD (median, 0.17% [IQR, −2.29% to 2.90%] vs 0.18% [−2.73% to 3.88%]) (*P* = .71). We observed a significant difference in the median LS-BMD percentage change for the ZOL subgroup with 3 or more years of denosumab treatment before enrollment (−3.20% [IQR, −7.89% to 0.68%]) compared with group A (1.30% [IQR, −0.68% to 5.24%]) (*P *= .003).

**Conclusions and Relevance:**

In this randomized trial of sequential therapy after denosumab discontinuation, bone loss was observed in LS-BMD in the first year among patients receiving zoledronate. A longer duration of denosumab treatment was associated with a further decrease in LS-BMD after zoledronate sequential therapy. Further randomized clinical trials and large-scale studies that investigate the strategies of sequential therapy after long-term denosumab treatment are needed.

**Trial Registration:**

ClinicalTrials.gov Identifier: NCT03868033

## Key Points

**Question:**

Does zoledronate prevent bone loss after denosumab discontinuation in the first year?

**Findings:**

In this randomized clinical trial of 101 participants who received denosumab for 2 or more years, sequential therapy with zoledronate did not prevent loss of bone mineral density in the lumbar spine (LS-BMD) in the first year. In multivariable and subgroup analyses, a longer duration of denosumab use was associated with further decreased LS-BMD; however, BMD in the femoral neck and total hip was preserved after sequential therapy.

**Meaning:**

These findings suggest that sequential therapy with zoledronate does not fully prevent a decrease in LS-BMD after denosumab discontinuation.

## Teaser

This randomized clinical trial examines the use of sequential therapy with zoledronate to prevent loss of bone mineral density after denosumab discontinuation in the first year.

## Introduction

Denosumab is an effective antiosteoporosis medication if the patient receives it on schedule.^1^ Abrupt discontinuation of denosumab, especially after prolonged treatment, leads to substantial bone loss and potentially increased risk of vertebral fractures.^2,3,4^ The efficacy of subsequent treatment with bisphosphonates after denosumab discontinuation to preserve gains in bone mineral density (BMD) has been examined in different studies and case series, with heterogeneous results.^5,6,7^ Two prospective randomized clinical trials reported inconsistent results about whether zoledronate prevents bone loss after discontinuation of denosumab treatment.^8,9^ We conducted this trial (1) to investigate whether zoledronate prevents bone loss 1 year after denosumab discontinuation and (2) to identify a better strategy for sequential therapy with zoledronate in the second year.

## Methods

### Trial Design and Registration

For the Denosumab Sequential Therapy (DST) prospective, open-label, parallel-group randomized clinical trial, recruitment was conducted from April 1, 2019, to May 31, 2021, and a 2-year follow-up was planned. The trial had 4 intervention groups, and it was conducted at 1 tertiary medical center (National Taiwan University Hospital [NTUH]) and 2 affiliated branches in separate regions in Taiwan (NTUH Hsinchu Branch and NTUH Yunlin Branch). The NTUH Research Ethics Committee approved the study. Patients provided written informed consent. The 2-year study protocol was published previously.^10^ In the first year, we aimed to investigate whether zoledronate prevented bone loss; in the second year, we tested 3 strategies of sequential therapy with a positive control group. Next, we describe the protocol mainly for the first year. The original protocol translated from Mandarin is provided in Supplement 1. The full study flow diagram is provided in the eFigure in Supplement 2. The study followed the Consolidated Standards of Reporting Trials (CONSORT) reporting guideline.

### Trial Population and Data Collection

This study enrolled postmenopausal women and men aged 50 years or older who were continuing regular denosumab (60 mg) treatment every 6 months for 2 or more years. The exclusion criteria were as follows: previous exposure to antiosteoporosis medications other than denosumab, an estimated glomerular filtration rate of less than 35 mL/min/1.73 m^2^, malignant neoplasm, ongoing medical treatment affecting bone metabolism, secondary osteoporosis, metabolic bone diseases, contraindication to zoledronate, age exceeding 80 years at the time of eligibility assessment, and hypocalcemia. After we obtained patient consent, baseline demographic characteristics, history of previous doses of denosumab, possible adverse effects after denosumab administration, past histories of fractures, and comorbidities were recorded. Baseline laboratory tests were conducted to assess serum levels of creatinine, album, calcium, serum N-terminal propeptide of type 1 collagen (P1NP), and C-terminal telopeptide (CTX) after patients fasted for at least 8 hours. Serum levels of bone turnover markers (BTMs) P1NP and CTX were tested biannually in the first year. All tests were analyzed at Union Clinical Laboratory using electrochemiluminescence immunoassays on a Cobas 411 analyzer (Roche Diagnostics), regardless of the recruitment center. Because serum levels of CTX and P1NP may increase after trauma, outliers of CTX and P1NP levels accompanied with known fracture or trauma-related immobilization were excluded.^11,12^

Baseline and 1-year BMD in the lumbar spine (LS-BMD), total hip (TH-BMD), and femoral neck (FN-BMD) were measured at each center by a certified bone densitometry technologist. Dual-energy x-ray absorptiometry (DXA) equipment used included the GE Lunar Prodigy (GE HealthCare) at NTUH and the Discovery Wi (Hologic) at both NTUH affiliated branches in Hsinchu and Yunlin. The calculated least significant change (LSC) ranged from 4.2% to 4.7% for LS-BMD, whereas the LSCs for TH-BMD and FN-BMD were close to but less than 4%. Therefore, we selected 5% as the LSC for LS-BMD and 4% for TH-BMD and FN-BMD, respectively. If the patient had not undergone prior hip surgery, the right hip was measured; otherwise, the left hip was measured. T scores were calculated based on the Asian reference population aged 20 to 40 years.^13^ The quality control program for DXA examinations ensured accuracy and reliability as delineated in the 2019 International Society for Clinical Densitometry position statement.^14^

Study data were collected and managed using REDCap (Research Electronic Data Capture) tools hosted at NTUH and its affiliated branches in Hsinchu and Yunlin.^15^

### Stratified Randomization and the Intervention

Participants were stratified by biological sex and T score (>−2.5 vs ≤−2.5) to 4 strata. Stratified participants were then randomly allocated to 4 intervention groups via a computer-generated sequence hidden from the investigators.^10^ Participants in group A received denosumab (60 mg) subcutaneously every 6 months on schedule throughout the 2-year study as the positive control. A negative control group was not possible due to ethical concerns. Participants in groups B, C, and D all received 1 dose of zoledronate (5 mg) intravenously, 6 months after their last dose of denosumab, during the first year of the study; these 3 groups were combined as group ZOL for this report. All participants were instructed to acquire at least 800 IU of vitamin D_3_ and 1000 mg of calcium daily.

### Outcomes and Sample Size Calculation

The coprimary outcomes of this study were the percentage changes in LS-BMD, TH-BMD, and FN-BMD among the study groups, assessed yearly. Secondary outcomes included the incidence of morphological and clinical osteoporotic vertebral fractures, the incidence of other osteoporotic fractures, and differences in BTM levels including P1NP and CTX. To detect a 2.6% decrease in TH-BMD while achieving 90% power and a 2-sided error α probability of 5% with an SD of 3.27%, a minimum of 19 patients were required in each group.^1,16^ The calculated sample size needed to detect bone loss in LS-BMD and FN-BMD was less than 19.^16,17^ We estimated that approximately 25 participants in each group would be adequate to account for potential dropouts. We kept enrollment and randomization open until each of the 4 groups had at least 25 participants.

Serum levels of P1NP and CTX were checked at baseline and at 6, 12, 15, 18, and 24 months after randomization.^18,19,20^ Vertebral fractures were confirmed annually by radiograph and as needed based on physician judgment. Other osteoporotic fractures were identified clinically. Adverse events reported by patients or observed by researchers were recorded.

### Statistical Analysis

In the first year of the trial, we aimed to explore the extent of bone loss after patients transitioned medication. Percentage changes in BMD of participants in group A served as the positive control. At the final follow-up of the second year, we will investigate percentage changes in BMD among the 4 groups.

An intention-to-treat analysis was performed. The Shapiro-Wilk test was used to examine normality. Percentage changes in BMD and changes in BTM serum levels were compared with the *t* test or Mann-Whitney *U* test, depending on normality.

We used several measures for the post hoc analysis. First, we separated participants who received zoledronate in the first year into 2 groups by the duration of denosumab treatment before they entered the trial. Individuals who had received denosumab treatment for 3 years or more were categorized into one subgroup, whereas those receiving a treatment duration of less than 3 years were placed in another group. We analyzed differences in percentage changes in LS-BMD, TH-BMD, and FN-BMD.

Second, we aimed to identify factors associated with bone loss more than the LSC at the LS after sequential therapy with zoledronate in the first year.^21^ Univariate and multivariable logistic regression analyses were performed using the variables listed in Table 1. Factors with *P* < .10 in the univariable logistic regression analysis were included in the final multivariable analysis. Finally, we examined the pattern of BTM levels regarding the duration of denosumab treatment. To address the potential inflation of type I error arising from multiple comparisons, we applied a Bonferroni correction, excluding primary outcomes. After applying the Bonferroni correction, the α level was adjusted to 0.006 (0.05/9 = 0.006) for secondary outcomes, subgroup analysis, and post hoc analysis. *P* < .05 (2-tailed) was considered statistically significant. We used R, version 4.0.4 (R Project for Statistical Computing), for all statistical analyses.

**Table 1.** Baseline Participant Demographics After Randomization^a^

Characteristic	Group A (n = 25)^b^	Group ZOL (n = 76)^c^
Biological sex		
Female	23 (92.0)	72 (94.7)
Male	2 (8.0)	4 (5.3)
Prior fracture	25 (100)	74 (97)
Previous vertebral fracture	21 (84)	66 (87)
Previous multiple vertebral fractures	11 (46)	41 (54)
Osteoporosis before trial enrollment	14 (56)	42 (55)
Denosumab treatment duration, y	2.0 (2.0 to 2.5)	2.0 (2.0 to 2.5)
Age, y	74.0 (70.0 to 78.0)	71.0 (65.7 to 76.0)
Height, cm	151 (147 to 157)	153 (148 to 155)
Weight, kg	57.0 (53.0 to 59.0)	56.0 (47.8 to 62.3)
BMI	24.4 (22.2 to 26.3)	24.1 (21.1 to 26.7)
T score		
Lumbar spine	−1.8 (−2.3 to −1.2)	−2.0 (−2.6 to −1.2)
Total hip	−1.8 (−2.0 to −1.4)	−1.5 (−2.0 to −1.0)
Femoral neck	−2.3 (−2.6 to −2.0)	−2.2 (−2.7 to −1.9)
Bone turnover marker serum level, ng/mL		
CTX	0.28 (0.19 to 0.38)	0.29 (0.15 to 0.43)
P1NP	26.0 (19.0 to 34.6)	23.7 (19.1 to 32.7)

Abbreviations: BMI, body mass index (calculated as weight in kilograms divided by height in meters squared); CTX, C-terminal telopeptide; P1NP, N-terminal propeptide of type 1 collagen; ZOL, zoledronate.

^a^ Values are presented as No. (%) of patients or median (IQR).

^b^ Group A received denosumab and served as the positive control.

^c^ Group ZOL comprises participants in groups B, C, and D, who all received zoledronate in the first year.

## Results

**Figure 1.**
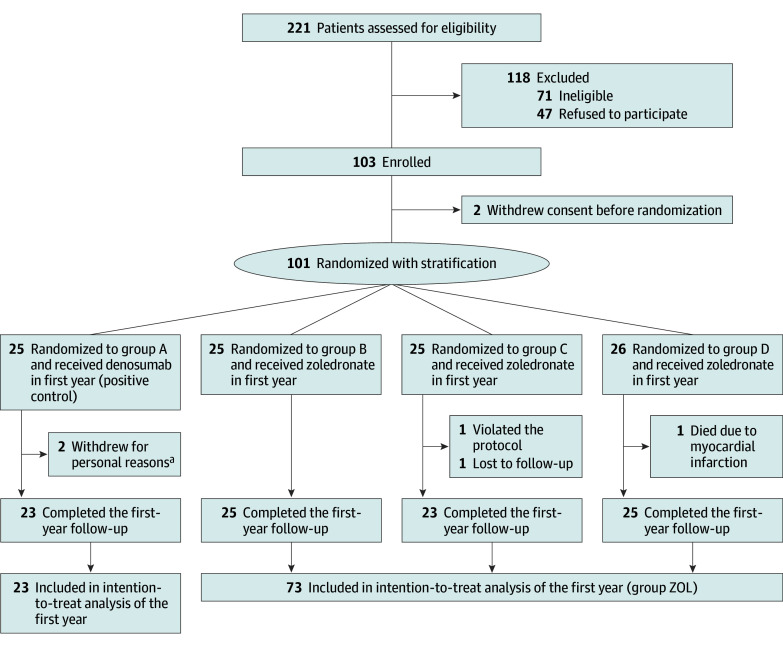
Flow Diagram of the First Year of the Trial ^a^One patient withdrew consent owing to noncompliance with the follow-up protocol; the other patient withdrew consent due to difficulty in drawing blood, which led to frustration after multiple attempts.

Of the 221 patients assessed for eligibility, 47 refused to participate, 71 were ineligible, and 2 withdrew informed consent before randomization. Therefore, 101 participants were enrolled in the DST trial and were assigned to 1 of 4 groups via stratified randomization. There were 95 women (94.1%) and 6 men (5.9%), and the median age of the cohort was 72.0 (IQR, 67.0-76.0) years. The results of stratified randomization are provided in eTable 1 in Supplement 2. There were 25 patients in group A (23 women [92.0%] and 2 men [8.0%]; median age, 74.0 [IQR, 70.0 to 78.0] years) and 76 in group ZOL (72 women [94.7%] and 4 men [5.3%]; median age, 71.0 [IQR, 65.7 to 76.0] years). Nearly all patients had fractures before entering the DST trial, which is likely attributable to the National Health Insurance Administration of Taiwan requirement that both low bone density and major osteoporotic fractures must be present for individuals to be eligible for reimbursement of antiosteoporosis medications.^22,23^ Demographic data for the 2 groups are presented in Table 1. The study flow diagram of the first year of the trial is shown in Figure 1.

### Primary Outcomes and Subgroup Analysis

**Figure 2.**
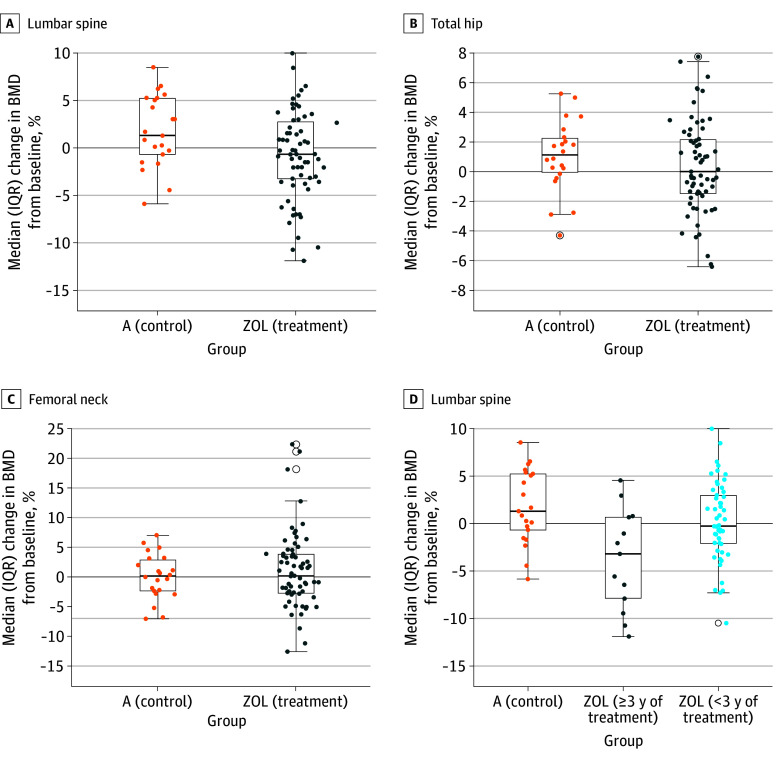
Changes in Bone Mineral Density (BMD) in the First Year of the Trial A, Compared with the positive control (group A), a significant difference in the percentage change in BMD after sequential therapy was observed in the lumbar spine for the zoledronate treatment group (group ZOL, which combines groups B, C, and D). B and C, No significant difference in the percentage change in BMD was observed for the total hip or femoral neck. D, Subgroup analysis suggested that the significant difference was associated with the duration of denosumab treatment before enrollment. A significant difference was only observed in the subgroup with 3 or more years of denosumab treatment before enrollment compared with group A and the ZOL subgroup with less than 3 years of treatment. Horizontal lines and boxes represent the median and IQR percentage changes in BMD, respectively; error bars represent 95% CIs.

At the end of the first year, a significant difference in the median percentage change in LS-BMD was noted between group A (1.30% [IQR, −0.68% to 5.24%]) and group ZOL (−0.68% [IQR, −3.22% to 2.75%]) (*P* = .03). No significant differences in the median percentage change between group A and group ZOL were observed for TH-BMD (1.12% [IQR, −0.06% to 2.25%] vs 0% [−1.47% to 2.15%]) (*P* = .24) and FN-BMD (0.17% [IQR, −2.29% to 2.90%] vs 0.18% [IQR, −2.73% to 3.88%]) (*P* = .71) (Figure 2A-C).

The 73 participants in group ZOL who received zoledronate and completed follow-up in the first year were further separated into 2 subgroups by the duration of denosumab treatment before they entered the trial. We observed a significant difference in the median LS-BMD percentage change for the ZOL subgroup with 3 or more years of denosumab treatment before enrollment (−3.20% [IQR, −7.89% to 0.68%]) compared with group A (1.30% [IQR, −0.68% to 5.24%]) (*P* = .003) (Figure 2D). In contrast, no significant difference was observed for the ZOL subgroup with less than 3 years of denosumab treatment (median, −0.28% [IQR, −2.11% to 2.97%]; *P* = .11) (Figure 2D). Between the 2 ZOL subgroups, no significant differences in LS-BMD percentage changes were observed after Bonferroni correction (Figure 2D). No significant differences in TH-BMD and FN-BMD were observed among group A (positive control) and the 2 ZOL subgroups. Details of BMD loss in the LS, TH, and FN exceeding the LSC, stratified by the duration of denosumab use, are summarized in eTable 2 in Supplement 2. Two participants in the overall cohort experienced significant BMD loss at the LS, TH, and FN. Changes in BMD among women who experienced vertebral fractures are reported in eTable 3 in Supplement 2. The results were similar to the main findings, due to the high prevalence of vertebral fractures in our study population.

We conducted a post hoc analysis of factors contributing to bone loss more than the LSC. On multivariable analysis, we observed that lower body weight and 3 or more years of denosumab treatment before medication transition contributed to bone loss more than the LSC at the LS (Table 2).

**Table 2.** Logistic Regression Analysis Results^a^

Possible risk factor (cutoff value)	Univariate analysis		Multivariate analysis	
	OR (95% CI)	*P* value	OR (95% CI)	*P* value
Age (72), y	0.44 (0.12-1.57)	.21	NA	NA
Height (153.0), cm	1.17 (0.33-4.13)	.81	NA	NA
Body weight (56.6), kg	6.00 (1.20-29.97)	.03	4.44 (1.04-18.96)	.04
BMI (18.5)	1.63 (0.29-9.30)	.58	NA	NA
VCF				
Prevalent	2.15 (0.25-18.82)	.49	NA	NA
Prior multiple	2.23 (0.60-8.23)	.23	NA	NA
Denosumab treatment duration, y				
≥2.5 vs <2.5	3.23 (0.89-11.75)	.08	NA	NA
≥3.0 vs <3.0	8.17 (1.99-33.58)	.004	6.91 (1.75-27.28)	.006
Bone turnover marker serum level, ng/mL				
CTX (0.30)	0.69 (0.20-2.44)	.56	NA	NA
P1NP (23.97)	0.37 (0.09-1.52)	.17	NA	NA
Osteoporosis before trial enrollment	0.45 (0.12-1.66)	.23	NA	NA

Abbreviations: BMI, body mass index (calculated as weight in kilograms divided by height in meters squared); CTX, C-terminal telopeptide; NA, not applicable; OR, odds ratio; P1NP, N-terminal propeptide of type 1 collagen; VCF, vertebral compression fracture.

^a^ Results are presented for the univariate analysis and the multivariable logistic regression using stepwise selection, which was conducted only for risk factors with *P* < .10 in the univariate analysis. With regard to denosumab treatment duration, there were 2 competing factors; only the more statistically significant factor was incorporated by the model.

### Secondary Outcomes and Subgroup Analyses

**Figure 3.**
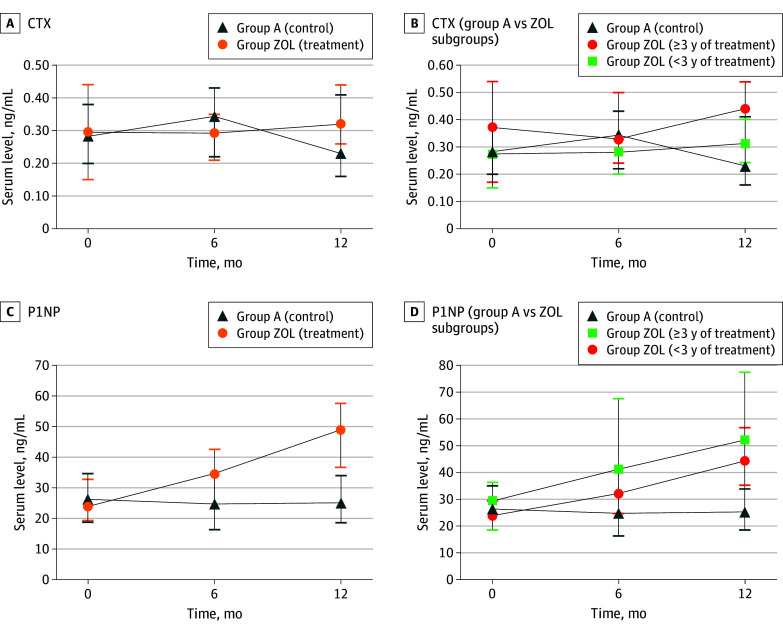
Serum Levels of Bone Turnover Markers A, At the end of the first year, there was no significant difference regarding C-terminal telopeptide (CTX) serum levels between the positive control (group A) and the treatment group (group ZOL, which combines groups B, C, and D). B, Compared with group A, the ZOL subgroup with 3 or more years of denosumab treatment at baseline had a significant difference in CTX serum levels at the end of the first year. No significant difference was observed for the ZOL subgroup with a shorter duration of denosumab treatment (<3 years). C, N-terminal propeptide of type 1 collagen (P1NP) serum levels increased significantly at the end of the first year in group ZOL. D, Both subgroups had significant differences in P1NP serum levels compared with group A.

After sequential therapy with zoledronate, there was no significant difference in median serum CTX at 1 year in group ZOL (0.32 [IQR, 0.26 to 0.44] ng/mL) compared with group A (0.23 [IQR, 0.16 to 0.41] ng/mL) (*P* = .07) (Figure 3A). Again, we separated patients who received sequential therapy into 2 subgroups by the duration of previous denosumab treatment. The median serum CTX in the ZOL subgroup with 3 or more years of denosumab treatment increased (0.44 [IQR, 0.30 to 0.54] ng/mL; *P* = .02), but this increase was not significant after Bonferroni correction compared with group A (positive control) (Figure 3B). There were no significant differences between median serum CTX of group A and patients with a shorter duration of previous denosumab treatment (0.31 [IQR, 0.24 to 0.40] ng/mL; *P* = .15).

The median serum P1NP of the ZOL subgroups after sequential therapy increased significantly at 1 year (48.9 [IQR, 36.7 to 57.5] ng/mL) compared with group A (25.1 [IQR, 18.5 to 33.9] ng/mL) (*P* < .001) (Figure 3C). Significant differences in median serum levels of P1NP were observed for both subgroups, regardless of the duration of previous denosumab treatment before enrollment (51.9 [IQR, 49.1 to 77.4] ng/mL for ≥3 years vs 44.2 [IQR, 35.3 to 56.6] ng/mL for <3 years) compared with group A (25.1 [IQR, 18.5 to 33.9] ng/mL) (*P* < .001) (Figure 3D).

In the first year of the study, neither atypical fracture nor jaw osteonecrosis was observed. Three vertebral fractures occurred in female patients in group ZOL, with 1 patient dropping out after receiving romosozumab at another hospital. Group A had no vertebral fractures, but 1 FN fracture was reported. One patient in group D died from acute myocardial infarction unrelated to the trial.

## Discussion

In this trial, a significant decrease in BMD after sequential therapy with zoledronate was observed only in the LS. Post hoc analysis indicated that early sequential therapy with zoledronate before the sixth dose of denosumab had similar effects on bone preservation as continuous denosumab treatment. After prolonged treatment with 3 or more years of denosumab, zoledronate did not fully prevent bone loss; this finding was similar to that of the prospective cohort study by Makras et al.^24^ Although the results of our subgroup analysis should be interpreted carefully, they may well explain the different results of 2 other prospective randomized clinical trials^8,9^ regarding zoledronate as sequential therapy to denosumab discontinuation. In the study conducted by Anastasilakis et al,^9^ the mean duration of denosumab treatment before zoledronate was 2.4 years, and successful preservation of bone mass by zoledronate was reported. For participants in the randomized clinical trial by Sølling et al,^8^ the mean duration of denosumab treatment ranged from 4.4 to 5.2 years. Sølling et al^8^ reported that zoledronate did not fully prevent bone loss. Emerging evidence has also suggested that a longer duration of denosumab treatment was associated with vertebral fractures and bone loss after sequential therapy to denosumab treatment.^3,4,5,25^ A clear cutoff has not been established. The results of our multivariable analysis suggest that sequential therapy with zoledronate before the third year of continuous denosumab treatment (ie, before the sixth dose of denosumab) was more likely to preserve bone mass.

In our study, no difference in serum BTM levels were observed at baseline between the positive control group and the groups receiving zoledronate. Among participants with shorter denosumab exposure before sequential therapy, an approximately 100% increase in P1NP level, relatively maintained low CTX level, and relatively preserved bone mass were observed after treatment with zoledronate. The overview of BTM changes in the first year among participants who received early sequential therapy seemed to be an attenuated version of the anabolic window. Whether we can use this anabolic window to help decrease BMD loss requires further study.

In previous studies, the annual incidence of vertebral fractures was 11.8% after discontinuation of denosumab, rising to 16.0% after more than 2 years of treatment.^4^ Sequential zoledronate reduced first-year fracture risk 3% to 4% in RCTs and retrospective studies.^5,6,8,9^ Of the 76 patients in the DST trial, 3 experienced vertebral fracture in the first year. The reported risk factors for new vertebral fracture after denosumab discontinuation include prevalent vertebral fractures, parental history of hip fractures, hip BMD at the time of treatment, BTM levels, treatment duration of denosumab, off-treatment intervals, and sequential treatment choice.^2,3,4,5,9,26^ Notably, prevalent vertebral fractures were consistently identified as a common risk factor across all studies. In our study, all 3 participants with post–sequential therapy vertebral fractures were women with prevalent vertebral fractures; 2 had been receiving denosumab for 4 or more years before medication transition. Risk mitigation strategies include ensuring adherence, following denosumab with bisphosphonates, monitoring BTM levels, and providing supplementary injections as necessary.^25,26,27^ Curtis et al^28^ reported a substantial advantage of denosumab over alendronate. Patients who continued receiving denosumab for longer periods experienced greater reductions in fracture risk.^28^ The clinical data suggested that adherence to denosumab ranged from 45.4% to 71.9% at 2 years.^22,29,30,31,32^ Some patients may discontinue denosumab treatment for reasons other than poor adherence or persistence, such as the need for dental treatment, consequences of the COVID-19 pandemic, cost issues, and others.^3,33^ When medication transition from denosumab is expected or when long-term denosumab treatment may not be suitable, earlier medication transition with potent sequential therapy should be considered.^6,8,9,25,34^

### Limitations

Our study had several limitations. First, we could not conduct a study using vertebral fractures as the primary end point due to the relatively small sample size. However, of the randomized clinical trials addressing bone loss after sequential therapy with zoledronate, our study had the largest sample size to date. Second, baseline serum levels of CTX in this trial were higher than those in previous RCTs.^8,9^ One reason is due to Taiwan’s health insurance regulations, which state that low bone density and major osteoporotic fractures must both be present to be eligible for coverage of antiresorptive agents^23^; otherwise, patients must pay out of pocket. As a result, osteoporosis treatment in Taiwan often begins at a later stage, with more severe disease status and potentially accompanied by multiple osteoporotic fractures. Third, we had few male participants, which reflected clinical conditions. Fourth, we were not able to evaluate the efficacy of zoledronate applied at different times after denosumab discontinuation, as done by Sølling et al.^8^ However, in that study, zoledronate administered 6 months after the last dose was considered the most attractive and effective option. Fifth, our sample size was estimated based on the highest number needed for a single primary outcome, which may be insufficient for 3 coprimary outcomes and could lead to potential type I error inflation when these outcomes are assessed simultaneously. Finally, all of our participants were Asian; therefore, further investigation is needed for generalizability of the findings.

## Conclusions

In this randomized clinical trial, a significant decrease in LS-BMD was observed in the first year after sequential therapy with zoledronate compared with continuous denosumab treatment. No significant differences in TH-BMD or FN-BMD were observed. Subgroup and post hoc analyses both suggested that a longer duration of continuous denosumab treatment before sequential therapy (specifically ≥3 years) negatively affected the efficacy of zoledronate in preserving bone mass. Early sequential therapy with zoledronate before the sixth dose of denosumab had noninferior outcomes for LS-BMD compared with continuous denosumab treatment. Further randomized clinical trials and large-scale studies that investigate the strategies of sequential therapy after long-term denosumab treatment are needed.

## Notes

Supplement 1.
Trial Protocol

Supplement 2. **eFigure.** Flowchart of the 2-Year Study **eTable 1.** Results of Stratified Randomization **eTable 2.** Bone Mineral Density (BMD) Loss in the Lumbar Spine, Total Hip, and Femoral Neck Exceeding the Least Significant Change in the Zoledronate (ZOL) Group **eTable 3.** Bone Mineral Density (BMD) Changes in Women Who Had Ever Suffered Vertebral Fractures in the Zoledronate (ZOL) Group (n = 63)

Supplement 3.
Data Sharing Statement

## References

[1] Bone HG, Wagman RB, Brandi ML, . 10 years of denosumab treatment in postmenopausal women with osteoporosis: results from the phase 3 randomised FREEDOM trial and open-label extension. Lancet Diabetes Endocrinol. 2017;5(7):513-523. doi:10.1016/S2213-8587(17)30138-9 28546097

[2] Cummings SR, Ferrari S, Eastell R, . Vertebral fractures after discontinuation of denosumab: a post hoc analysis of the randomized placebo-controlled FREEDOM trial and its extension. J Bone Miner Res. 2018;33(2):190-198. doi:10.1002/jbmr.3337 29105841

[3] Fu SH, Wang CY, Hung CC, . Increased fracture risk after discontinuation of anti-osteoporosis medications among hip fracture patients: a population-based cohort study. J Intern Med. 2021;290(6):1194-1205. doi:10.1111/joim.13354 34237171

[4] Cosman F, Huang S, McDermott M, Cummings SR. Multiple vertebral fractures after denosumab discontinuation: FREEDOM and FREEDOM Extension trials additional post hoc analyses. J Bone Miner Res. 2022;37(11):2112-2120. doi:10.1002/jbmr.4705 36088628 PMC10092421

[5] Everts-Graber J, Reichenbach S, Gahl B, Ziswiler HR, Studer U, Lehmann T. Risk factors for vertebral fractures and bone loss after denosumab discontinuation: a real-world observational study. Bone. 2021;144:115830. doi:10.1016/j.bone.2020.115830 33359006

[6] Tutaworn T, Nieves JW, Wang Z, Levin JE, Yoo JE, Lane JM. Bone loss after denosumab discontinuation is prevented by alendronate and zoledronic acid but not risedronate: a retrospective study. Osteoporos Int. 2023;34(3):573-584. doi:10.1007/s00198-022-06648-9 36602607 PMC9813893

[7] Reid IR, Horne AM, Mihov B, Gamble GD. Bone loss after denosumab: only partial protection with zoledronate. Calcif Tissue Int. 2017;101(4):371-374. doi:10.1007/s00223-017-0288-x 28500448

[8] Sølling AS, Harsløf T, Langdahl B. Treatment with zoledronate subsequent to denosumab in osteoporosis: a 2-year randomized study. J Bone Miner Res. 2021;36(7):1245-1254. doi:10.1002/jbmr.4305 33813753

[9] Anastasilakis AD, Papapoulos SE, Polyzos SA, Appelman-Dijkstra NM, Makras P. Zoledronate for the prevention of bone loss in women discontinuing denosumab treatment: a prospective 2-year clinical trial. J Bone Miner Res. 2019;34(12):2220-2228. doi:10.1002/jbmr.3853 31433518

[10] Lee CC, Wang CY, Hung CC, . A multi-institutional randomized controlled trial to investigate whether zoledronate prevents bone loss after discontinuation of denosumab: the study protocol of Denosumab Sequential Therapy (DST) trial. Front Med (Lausanne). 2021;8:717168. doi:10.3389/fmed.2021.717168 34568375 PMC8455904

[11] Stewart CC, O’Hara NN, Bzovsky S, Bahney CS, Sprague S, Slobogean GP; Vita-Shock Investigators. Bone turnover markers as surrogates of fracture healing after intramedullary fixation of tibia and femur fractures. Bone Joint Res. 2022;11(4):239-250. doi:10.1302/2046-3758.114.BJR-2021-0226.R1 35442058 PMC9057525

[12] Heer M, Baecker N, Mika C, Boese A, Gerzer R. Immobilization induces a very rapid increase in osteoclast activity. Acta Astronaut. 2005;57(1):31-36. doi:10.1016/j.actaastro.2004.12.007 15900645

[13] Lu YC, Lin YC, Lin YK, . Prevalence of osteoporosis and low bone mass in older Chinese population based on bone mineral density at multiple skeletal sites. Sci Rep. 2016;6:25206. doi:10.1038/srep25206 27143609 PMC4855183

[14] Shuhart CR, Yeap SS, Anderson PA, . Executive summary of the 2019 ISCD Position Development Conference on monitoring treatment, DXA cross-calibration and least significant change, spinal cord injury, peri-prosthetic and orthopedic bone health, transgender medicine, and pediatrics. J Clin Densitom. 2019;22(4):453-471. doi:10.1016/j.jocd.2019.07.001 31400968

[15] Harris PA, Taylor R, Minor BL, ; REDCap Consortium. The REDCap Consortium: building an international community of software platform partners. J Biomed Inform. 2019;95:103208. doi:10.1016/j.jbi.2019.103208 31078660 PMC7254481

[16] Lehmann T, Aeberli D. Possible protective effect of switching from denosumab to zoledronic acid on vertebral fractures. Osteoporos Int. 2017;28(10):3067-3068. doi:10.1007/s00198-017-4108-y 28589418

[17] Popp AW, Varathan N, Buffat H, Senn C, Perrelet R, Lippuner K. Bone mineral density changes after 1 year of denosumab discontinuation in postmenopausal women with long-term denosumab treatment for osteoporosis. Calcif Tissue Int. 2018;103(1):50-54. doi:10.1007/s00223-018-0394-4 29380013

[18] Michelsen J, Wallaschofski H, Friedrich N, . Reference intervals for serum concentrations of three bone turnover markers for men and women. Bone. 2013;57(2):399-404. doi:10.1016/j.bone.2013.09.010 24076251

[19] Eastell R, Szulc P. Use of bone turnover markers in postmenopausal osteoporosis. Lancet Diabetes Endocrinol. 2017;5(11):908-923. doi:10.1016/S2213-8587(17)30184-5 28689768

[20] Li M, Li Y, Deng W, . Chinese bone turnover marker study: reference ranges for C-terminal telopeptide of type I collagen and procollagen I N-terminal peptide by age and gender. PLoS One. 2014;9(8):e103841. doi:10.1371/journal.pone.0103841 25117452 PMC4130521

[21] Diez-Perez A, Adachi JD, Agnusdei D, ; IOF CSA Inadequate Responders Working Group. Treatment failure in osteoporosis. Osteoporos Int. 2012;23(12):2769-2774. doi:10.1007/s00198-012-2093-8 22836278

[22] Lee CC, Fu SH, Chen HM, . The real-world adherence of the first-line anti-osteoporosis medications in Taiwan: visualize the gap between reality and expectations. J Formos Med Assoc. 2023;122(suppl 1):S55-S64. doi:10.1016/j.jfma.2023.05.022 37302970

[23] Wang CY, Fu SH, Hung CC, . Impact of the requirement of bone mineral density evidence on utilization of anti-osteoporosis medications, clinical outcome and medical expenditures of patient with hip fracture in Taiwan. Int J Health Policy Manag. 2022;11(4):470-478. 33059424 10.34172/ijhpm.2020.169 PMC9309953

[24] Makras P, Appelman-Dijkstra NM, Papapoulos SE, . The duration of denosumab treatment and the efficacy of zoledronate to preserve bone mineral density after its discontinuation. J Clin Endocrinol Metab. 2021;106(10):e4155-e4162. doi:10.1210/clinem/dgab321 33978745

[25] Tsourdi E, Zillikens MC, Meier C, . Fracture risk and management of discontinuation of denosumab therapy: a systematic review and position statement by ECTS. J Clin Endocrinol Metab. 2021;106(1):264-281. doi:10.1210/clinem/dgaa756 33103722

[26] Burckhardt P, Faouzi M, Buclin T, Lamy O; the Swiss Denosumab Study Group. Fractures after denosumab discontinuation: a retrospective study of 797 cases. J Bone Miner Res. 2021;36(9):1717-1728. doi:10.1002/jbmr.4335 34009703 PMC8518625

[27] Reid IR, Billington EO. Drug therapy for osteoporosis in older adults. Lancet. 2022;399(10329):1080-1092. doi:10.1016/S0140-6736(21)02646-5 35279261

[28] Curtis JR, Arora T, Liu Y, . Comparative effectiveness of denosumab vs alendronate among postmenopausal women with osteoporosis. J Bone Miner Res. 2024;39(7):826-834. doi:10.1093/jbmr/zjae079 38753892 PMC11301726

[29] Pedersen AB, Risbo N, Kafatos G, Neasham D, O’Kelly J, Ehrenstein V. Utilization patterns and factors associated with persistence of new users of anti-osteoporosis treatment in Denmark: a population-based cohort study. Arch Osteoporos. 2023;18(1):19. doi:10.1007/s11657-023-01210-4 36629929 PMC9834110

[30] Reyes C, Tebe C, Martinez-Laguna D, . One and two-year persistence with different anti-osteoporosis medications: a retrospective cohort study. Osteoporos Int. 2017;28(10):2997-3004. doi:10.1007/s00198-017-4144-7 28714038

[31] Silverman SL, Siris E, Belazi D, . Persistence at 24 months with denosumab among postmenopausal women with osteoporosis: results of a prospective cohort study. Arch Osteoporos. 2018;13(1):85. doi:10.1007/s11657-018-0491-z 30088189 PMC6096691

[32] Yazan CD, Bugdayci O, Ilgin C, Yavuz DG. Effect of denosumab treatment on bone mineral density and bone turnover markers in osteoporotic patients: real-life experience 2-year follow-up. Arch Osteoporos. 2022;17(1):125. doi:10.1007/s11657-022-01145-2 36114901

[33] Hong N, Shin S, Lee S, Kim KJ, Rhee Y. Raloxifene use after denosumab discontinuation partially attenuates bone loss in the lumbar spine in postmenopausal osteoporosis. Calcif Tissue Int. 2022;111(1):47-55. doi:10.1007/s00223-022-00962-4 35226133

[34] Ebina K, Tsuboi H, Nagayama Y, . Effects of prior osteoporosis treatment on 12-month treatment response of romosozumab in patients with postmenopausal osteoporosis. Joint Bone Spine. 2021;88(5):105219. doi:10.1016/j.jbspin.2021.105219 34020048

