# Supplementary material for: Zoledronate Sequential Therapy After Denosumab Discontinuation to Prevent Bone Mineral Density Reduction: A Randomized Clinical Trial
Source: JAMA Netw Open. 2024 Nov 11;7(11):e2443899. doi: 10.1001/jamanetworkopen.2024.43899 (PMC11555552; doi:10.1001/jamanetworkopen.2024.43899)
Supplement: Supplement 1. — Trial Protocol [file jamanetwopen-e2443899-s001.pdf]

Research Proposal of National Taiwan University Hospital

1. Chinese Project Title

以 Zoledronic acid 作為 Denosumab 的接續治療---是否能有效避免 Denosumab 停藥後快速的骨質流失?

2. English Project Title

Sequential therapy after Denosumab by Zoledronic acid --- could bone loss post discontinuation of Denosumab be prevented?

3. Principal Investigator：Shau-Huai, Fu (National Taiwan University Hospital, Yunlin branch)

Co-Principal Investigator：Chuan-Ching Huang (National Taiwan University Hospital, Yunlin branch), Chih-Chien Hung (National Taiwan University Hospital, Yunlin branch), Chia-Che Lee (National Taiwan University Hospital), Hsuan-Yu Chen (National Taiwan University Hospital, Hsinchu branch)

4. Research Topic Description

Denosumab is a human monoclonal antibody that inhibits Receptor Activator of Nuclear Factor Kappa B Ligand (RANKL), resulting in an anti-resorptive effect. Since its market introduction, it has gained increasing market share in Taiwan[2] due to its simplicity (subcutaneous injection) and less frequent dosing (once every six months). Patient adherence and persistence with treatment are higher compared to traditional bisphosphonate medications[1]. Although its preventive effects on fractures and increase in bone density have demonstrated excellent performance over a follow-up period of up to ten years[3], and with minimal side effects, related studies indicate that the anti-resorptive effect of denosumab is reversible. After discontinuation, patients experience a rapid decline in bone density, sometimes returning to pre-treatment levels within just one year[4]. In addition to the rapid decline in bone density, there have been numerous case reports indicating that discontinuation of denosumab may lead to severe rebound effects and multiple vertebral compression fractures[6-8]. However, based on previous research, despite higher persistence with treatment for patients receiving

Prolia (Denosumab) compared to traditional oral bisphosphonates, with only 62% drug adherence two years into treatment[5], the long-term efficacy of Denosumab is compromised in the context of decreasing medication adherence. Therefore, how to effectively consolidate the increased bone density after a period of Denosumab treatment, preventing rapid bone density loss due to factors such as poor adherence, persistence, or other health-related issues, becomes an important and unresolved issue.

The current research on the post-discontinuation consolidation effects of Denosumab treatment remains quite limited. In 2012, Freemantle et al. conducted a study indicating that in a group switching from Prolia (Denosumab) to Fosamax (Alendronate) for one year after one year of Prolia use, bone turnover markers slightly increased, but bone density did not significantly decrease, and even remained stable[1]. However, the primary focus of that study was on adherence and persistence, with a relatively short duration of only one year. Additionally, the chosen medication was a less potent oral bisphosphonate, leaving the aforementioned issues still requiring further investigation. In 2015, Leder et al. reported findings from the DATA-Switch study, revealing that if patients switch from 2 years of Denosumab to 2 years of Teriparatide treatment, a decrease in bone density was observed in the hip and wrist regions compared to values after 2 years of Denosumab treatment. Simultaneously, relevant bone turnover markers increased[9]. However, due to restrictions on Teriparatide reimbursement by the Taiwan National Health Insurance Bureau, limited to patients with later-stage fractures, and the overall higher cost of treatment compared to anti-resorptive drugs, discussing the continuity of Denosumab with Teriparatide is not practical in the routine clinical setting in Taiwan. Furthermore, among anti-resorptive drugs, the mechanisms of action differ between bisphosphonates, which directly target osteoclasts, and the monoclonal antibody Denosumab. Past studies have shown that bisphosphonates maintain bone density effectively after discontinuation[10,11], with Zoledronic acid, due to its long duration of action and a dosing interval of up to 1 year, mitigating the impact of patient adherence. Additionally, Zoledronic acid can effectively inhibit bone turnover markers even six months after discontinuation, maintaining bone density[12]. Some case studies suggest that using Zoledronic acid as a follow-up treatment to Denosumab may partially protect against rapid bone loss[13,14]. The study aims to conduct a randomized controlled trial to examine whether using Zoledronic acid as a follow-up treatment to Denosumab can prevent rapid bone loss after discontinuation of Denosumab.

5. Study Objectives

This study aims to investigate whether using Zoledronic acid (Aclasta) after discontinuation of Prolia (Denosumab) can prevent bone loss and avoid increased bone metabolism.

Primary outcome:

1. Changes in lumbar spine, total hip and femoral neck bone mineral density (BMD) rom baseline to one year and two years

Secondary outcomes:

1. Changes of C-terminal telopeptide of type I collagen (CTX)
2. Changes of propeptide of procollagen type I (P1NP)
3. Incidence of clinical osteoporotic fracture

6. Research Methods and Procedures

6.1. Subject Selection Criteria.

Inclusion criteria:

Post-menopausal women and men aged 50 years and over, who had consistently received biannual denosumab treatment for at least two years

Exclusion criteria:

- Secondary osteoporosis
- Metabolic bone diseases
- Cancer
- Ongoing steroid therapy
- Hormone replacement therapy
- Current use of any medications affecting bone metabolism
- Prior use of any non-denosumab osteoporosis treatment
- Glomerular filtration rate less than <35 mL/min
- Allergy to zoledronic acid
- Low blood calcium
- Any other contraindications to the use of zoledronic acid
- Age greater than 80 years

6.2. Assessment Items

6.2.1 Basic Information:

Age, gender, height, weight, medical history, fracture history, original bone density examination records.

6.2.2 Clinical Records:

Any side effects and newly occurring fractures during the follow-up treatment period.

6.2.3 Blood Tests:

C-terminal telopeptide of type I collagen (CTX) & Propeptide of procollagen type I (P1NP): Blood samples will be collected for bone turnover markers (CTX) and bone formation markers (P1NP) at the start of the study and subsequently at six months, one year, one year three months, one year six months, and two years, totaling six times. Approximately 5ml of blood will be drawn each time.

6.2.4 Bone Density Examination:

Bone density will be examined at the beginning of the study and then at one year and two years after medication, totaling three times. The radiation dose for each bone density examination is approximately 1-5 microsieverts.

6.2.5 Newly Occurring Fracture Events During the Follow-up Period:

Fracture is defined as any fracture excluding those in the hands, feet, nose, and skull.

6.3. Study Procedures

(1) Identify suitable participants based on inclusion and exclusion criteria. After providing detailed explanations, inquire about their willingness to participate in the study. If agreed, have them sign the informed consent form (as attached).

(2) Perform stratified random allocation to determine four groups as illustrated in the diagram below.

(3) Collect blood samples for CTX & P1NP testing before medication, and at six months, one year, one year three months, one year six months, and two years after medication.

(4) Examine follow-up bone density before medication, and at one year and two years after medication.

(5) Record any side effects and newly occurring fractures during the follow-up treatment period.

(6) Assist in supplementing adequate calcium (800-1000mg/day) and vitamin D (800 IU/day) during the follow-up period.

(7) In case of severe adverse reactions during the study, the participant will terminate the trial, but monitoring will continue.

For detailed enrollment procedures, please refer to the diagram below:

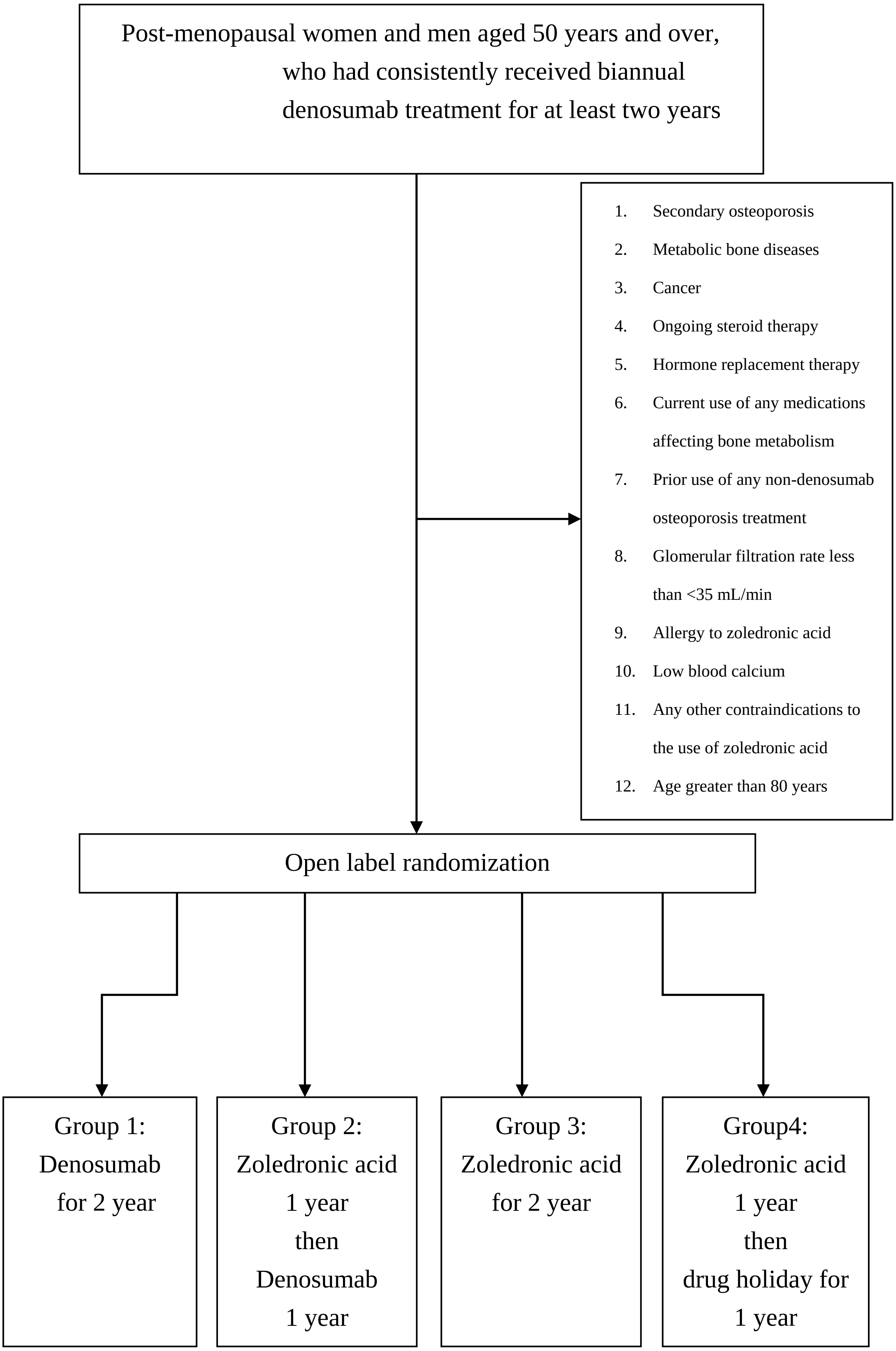

Study flow chart

6.4 Statistical Analysis

6.4.1 Randomization:

This study will employ a stratified randomization method to avoid potential uneven distribution of certain characteristics due to a smaller sample size. Patients will be stratified based on sex and bone mineral density (the lowest T-score (>-2.5 or ≤-2.5)). Randomization will then be conducted within each stratum. The four groups in this trial will be randomized in a 1:1:1:1 ratio.

6.4.2 Duration of Study Outcome Follow-up:

(1) BMD of lumbar spine, total hip and femoral neck will be measured at baseline, 12 months and 24 months.

(2) CTX and P1NP will be measured at baseline, 6 months, 12 months, 15 months, 18 months, and 24 months.

(3) Subsequent fracture will be recorded during entire follow-up period.

Please refer to the table below for the complete follow-up duration.

	Baseline	6m	12m	15m	18m	24m
Basic information	✓					
Bone mineral density	✓		✓			✓
Bone turnover marker	✓	✓	✓	✓	✓	✓
New fracture	Reporting and documentation are allowed throughout the entire follow-up period.					
Side effect	Reporting and documentation are allowed throughout the entire follow-up period.					

6.4.3 Dynamic Monitoring and Adjustment of Treatment Strategy for Group Four

Group Four is designed to observe whether Zoledronic acid can continuously prevent bone loss after discontinuation of Denosumab. Current guidelines suggest reevaluation of bisphosphonate drugs (including Zoledronic acid) after three to five years of treatment, with consideration of a drug holiday for low-risk patients. However, there is no consensus on the duration of Denosumab treatment. Therefore, this group is set to observe the cessation of treatment after two years of Denosumab and one year of Zoledronic acid therapy. Previous literature has indicated that the therapeutic effects of

Zoledronic acid can last for over 18 months. Hence, after discontinuation, in addition to continued supplementation of adequate vitamin D and calcium, bone turnover markers will be actively monitored. If the bone turnover markers exceed the normal concentrations for typical osteoporosis patients (reference values: postmenopausal osteoporotic women >0.573 ng/mL; osteoporotic men >0.584 ng/mL), reinforcement with a dose of Zoledronic acid will be administered.

6.4.4 Descriptive Statistics

Describe the basic characteristic differences among the four study groups. Chi-square tests will be used for categorical variables, while one-way analysis of variance (ANOVA) will be used for numerical variables to determine if there are differences among the groups.

Statistical Analysis of Research Results:
(1) Present the average Bone Mineral Density (BMD) values within each subgroup at baseline, 12, and 24 months. Utilize one-way ANOVA to investigate whether there are statistically significant differences in BMD changes (proportions) among the groups. Additionally, if one-way ANOVA indicates significant differences between groups, independent t-tests will be performed for pairwise comparisons to identify which two groups exhibit significant differences. Given that BMD change values may not follow a normal distribution, Kruskal-Wallis and Wilcoxon rank-sum tests will also be employed for this analysis.

(2) Display the average C-terminal telopeptide of type I collagen (CTX) and Propeptide of procollagen type I (P1NP) values within each subgroup at baseline, 6, 12, 15, 18, and 24 months. Use one-way ANOVA to examine whether there are statistically significant differences in changes among the groups. Similarly, if one-way ANOVA indicates significant differences between groups, independent t-tests will be conducted for pairwise comparisons. Given that hormonal change values may not follow a normal distribution, Kruskal-Wallis and Wilcoxon rank-sum tests will be employed for this analysis. Finally, create line graphs illustrating the long-term changes in CTX and P1NP within each group and use the Chow test to assess whether there are significant differences in long-term trends among the groups.

(3) Record the incidence of new fractures during the follow-up period using accumulated incidence rate. Analyze differences in incidence rates among the groups using the log-rank test.

(4) This study is a two-tailed test, and the significance level is set at 0.05.

7. Study Period and Progress (anticipated):

2019.04~2023.12

8. Names and Quantities of Required Medications or Medical Devices:

- Prolia® (Denosumab) injection (Amgen): Two doses per year
- Aclasta® (Zoledronic acid) injection 5mg/100ml (Novartis): One dose per year

9. Adverse Reactions and Handling in Clinical Setting:

Prolia (Denosumab): Possible adverse reactions in clinical settings include back pain, limb pain, musculoskeletal pain, hypocalcemia, severe infections, skin reactions, jaw necrosis, and atypical femoral fractures. Participants recruited for this study have already undergone two years of Prolia (Denosumab) treatment, making them more tolerant to the drug and reducing the likelihood of common adverse reactions.

Aclasta (Zoledronic acid): Possible adverse reactions in clinical settings include musculoskeletal pain, hypocalcemia, jaw necrosis, and atypical femoral fractures. Participants recruited for this study have only received one year of Aclasta (Zoledronic acid) treatment, reducing the likelihood of severe adverse reactions such as jaw necrosis and atypical femoral fractures.

10. Potential Risks and Benefits to Physical and Mental Health:

The medications used in this study are approved by the Ministry of Health and Welfare for the treatment of osteoporosis. Their safety has been tested globally for many years, and the risks mentioned above are relatively low compared to their effectiveness.

This study provides funding for tracking bone turnover markers and bone mineral density, enabling participants to monitor changes in treatment effects during the therapy period. This information serves as a basis for subsequent follow-up and treatment decisions.

11. Confidentiality and Privacy Protection of Participant Data:

Relevant data from this research project will be encrypted and stored in the Orthopedic Department of our institution. The principal investigator will be responsible for destroying the data after the research findings are published.

12. Conflict of Interest:

This study is self-initiated by the researchers and has no sponsorship from any company.

13. Research Personnel and Equipment:

Personnel: To be hired.

Equipment: Outpatient space at National Taiwan University Hospital, Yunlin Branch, Hsinchu branch and the imaging department's bone density examination room.

14. Funding Requirements:

Project Name	Purpose	Total Cost
Personnel Expenses		
Salary for Part-time Assistants	NT$480,000 (NT$10,000/month for a master's level part-time assistant, for 4 years)<br>Labor and Health Insurance Costs (906+794+666)124 = NT$113,568	593,568
Expert Meetings	NT$2,000 x (4 experts) x 4 sessions = NT$32,000<br>Unit Supplementary Premium 1.91%: NT$32,000 x 1.91% = NT$612	32,612
Temporary Wages	Wages for specific tasks required for the implementation of this project, limited to hourly pay. Calculated based on the latest hourly minimum wage announced by the Ministry of Labor, NT$120 per hour. Estimated at NT$960 per person-day x 70 person-days = NT$68,160	68,160
Participants' Travel Allowance	Compensation for the time and transportation expenses incurred by participants in the experiment. Estimated at 500 person-times (100 participants x 5 times) x NT$200 = NT$100,000	100,000
Bone Density Examination Fee	NT$600 per person x 100 persons x 2 times = NT$120,000	120,000
Bone Turnover Marker Test Fee	NT$1,700 per person x 100 persons x 3 times = NT$510,000	510,000
Stationery paper	Expenses for ink, paper, stationery, toner, and other supplies required for the implementation of this project.	30,000
Printing:	Printing and binding fees for books, research reports, professional education training materials, and photocopying expenses needed for the implementation of this project.	40,000

Total	1,494,340	

Total (anticipated):NT$ 1,494,340 元

15. Ownership and Utilization of Research and Development Results:

The ownership of the research outcomes belongs to National Taiwan University Hospital Yunlin Branch. In addition to serving as crucial reference material for future clinical treatments, the research team will also compile relevant results into papers for presentation at international conferences and publication in academic journals.

16. Reference：

1. Freemantle N, Satram-Hoang S, Tang ET, Kaur P, Macarios D, Siddhanti S, Borenstein J, Kendler DL; DAPS Investigators. Final results of the DAPS (Denosumab Adherence Preference Satisfaction) study: a 24-month, randomized, crossover comparison with alendronate in postmenopausal women. Osteoporos Int. 2012 Jan;23(1):317-26.
2. Wang CY, Shau-Huai Fu, Rong-Sen Yang, Fe-Lin Lin Wu, Fei-Yuan Hsiao. Epidemiology and Economic Burden of Osteoporosis in Taiwanese Women: A Nationwide Study, 2009-2013. 7th Asian Association of Schools of Pharmacy (AASP) Conference in Taipei Taiwan, on October 30- November 1, 2015.
3. Henry G Bone, Rachel B Wagman, Maria L Brandi, Jacques P Brown, Roland Chapurlat, Steven R Cummings, Edward Czerwiński, Astrid Fahrleitner-Pammer, David L Kendler, Kurt Lippuner, Jean-Yves Reginster, Christian Roux, Jorge Malouf, Michelle N Bradley, Nadia S Daizadeh, Andrea Wang, Paula Dakin, Nicola Pannacciulli, David W Dempster, Socrates Papapoulos. 10 years of denosumab treatment in postmenopausal women with osteoporosis: results from the phase 3 randomised FREEDOM trial and open-label extension Lancet Diabetes Endocrinol. 2017 Jul;5(7):513-523.
4. Bone HG, Bolognese MA, Yuen CK, Kendler DL, Miller PD, Yang YC, Grazette L, San Martin J, Gallagher JC. Effects of denosumab treatment and discontinuation on bone mineral density and bone turnover markers in postmenopausal women with low bone mass. J Clin Endocrinol Metab. 2011 Apr;96(4):972-80.
5. Karlsson L, Lundkvist J, Psachoulia E, Intorcia M, Ström O. Persistence with denosumab and persistence with oral bisphosphonates for the treatment of postmenopausal osteoporosis: a retrospective, observational study, and a meta-analysis. Osteoporos Int. 2015 Oct;26(10):2401-11.

6. Anastasilakis AD, Makras P, Multiple clinical vertebral fractures following denosumab discontinuation. Osteoporos Int 27 (5) (2016) 1929-1930.
7. Aubry-Rozier B, Gonzalez-Rodriguez E, Stoll D, Lamy O, Severe spontaneous vertebral fractures after denosumab discontinuation: three case reports, Osteoporos Int 27 (5) (2016) 1923-1925.
8. Popp AW, Zysset PK, Lippuner K, Rebound-associated vertebral fractures after discontinuation of denosumab-from clinic and biomechanics, Osteoporos Int 27 (5) (2016) 1917-1921.
9. Leder BZ, Tsai JN, Uihlein AV, Wallace PM, Lee H, Neer RM et al. Denosumab and teriparatide transitions in postmenopausal osteoporosis (the DATA-Switch study): extension of a randomised controlled trial. The Lancet 2015;386(9999):1147-55.
10. Black DM1, Reid IR, Boonen S, Bucci-Rechtweg C, Cauley JA, Cosman F, Cummings SR, Hue TF, Lippuner K, Lakatos P, Leung PC, Man Z, Martinez RL, Tan M, Ruzycky ME, Su G, Eastell R. The effect of 3 versus 6 years of zoledronic acid treatment of osteoporosis: a randomized extension to the HORIZON-Pivotal Fracture Trial (PFT). J Bone Miner Res. 2012 Feb;27(2):243-54.
11. Black DM1, Reid IR, Cauley JA, Cosman F, Leung PC, Lakatos P, Lippuner K, Cummings SR, Hue TF, Mukhopadhyay A, Tan M, Aftring RP, Eastell R. The effect of 6 versus 9 years of zoledronic acid treatment in osteoporosis: a randomized second extension to the HORIZON-Pivotal Fracture Trial (PFT). J Bone Miner Res. 2015 May;30(5):934-44.
12. V. Z. C. Borba, G. Paz-Filho, C. A. M. Kulak, M. J. Seibe,J. P. Bilezikian. Bone turnover 18 months after a single intravenous dose of zoledronic acid. Int J Clin Pract. 2007 Jun;61(6):1058-62.
13. Lehmann T, Aeberli D. Possible protective effect of switching from denosumab to zoledronic acid on vertebral fractures. Osteoporos Int. 2017 Oct;28(10):3067-3068.
14. Reid IR, Horne AM, Mihov B, Gamble GD. Bone Loss After Denosumab: Only Partial Protection with Zoledronate. Calcif Tissue Int. 2017 Oct;101(4):371-374.
15. Adler RA, El-Hajj Fuleihan G, Bauer DC, Camacho PM, Clarke BL, Clines GA, Compston JE, Drake MT, Edwards BJ, Favus MJ, Greenspan SL, McKinney R Jr, Pignolo RJ, Sellmeyer DE. Managing Osteoporosis in Patients on Long-Term Bisphosphonate Treatment: Report of a Task Force of the American Society for Bone and Mineral Research. J Bone Miner Res. 2016 Jan;31(1):16-35.
