# Supplementary material for: Zoledronate Sequential Therapy After Denosumab Discontinuation to Prevent Bone Mineral Density Reduction: A Randomized Clinical Trial
Source: JAMA Netw Open. 2024 Nov 11;7(11):e2443899. doi: 10.1001/jamanetworkopen.2024.43899 (PMC11555552; doi:10.1001/jamanetworkopen.2024.43899)
Supplement: Supplement 2. — eFigure. Flowchart of the 2-Year Study eTable 1. Results of Stratified Randomization eTable 2. Bone Mineral Density (BMD) Loss in the Lumbar Spine, Total Hip, and Femoral Neck Exceeding the Least Significant Change in the Zoledronate (ZOL) Group eTable 3. Bone Mineral Density (BMD) Changes in Women Who Had Ever Suffered Vertebral Fractures in the Zoledronate (ZOL) Group (n = 63) [file jamanetwopen-e2443899-s002.pdf]

Supplementary Online Content

Lee CC, Wang CY, Yen HK, et al. Zoledronate sequential therapy after denosumab discontinuation to prevent bone mineral density reduction: a randomized clinical trial. *JAMA Netw Open*. 2024;7(11):e2443899. doi:10.1001/jamanetworkopen.2024.43899

eFigure. Flow Chart of the 2-Year Study

eTable 1. Results of Stratified Randomization

eTable 2. Bone Mineral Density (BMD) Loss in the Lumbar Spine, Total Hip, and Femoral Neck Exceeding the Least Significant Change in the Zoledronate (ZOL) Group

eTable 3. Bone Mineral Density (BMD) Changes in Women Who Had Ever Suffered Vertebral Fractures in the Zoledronate (ZOL) Group (n = 63)

This supplementary material has been provided by the authors to give readers additional information about their work.

© 2024 Lee CC et al. *JAMA Network Open.*

eFigure. Flow Chart of the 2-Year Study

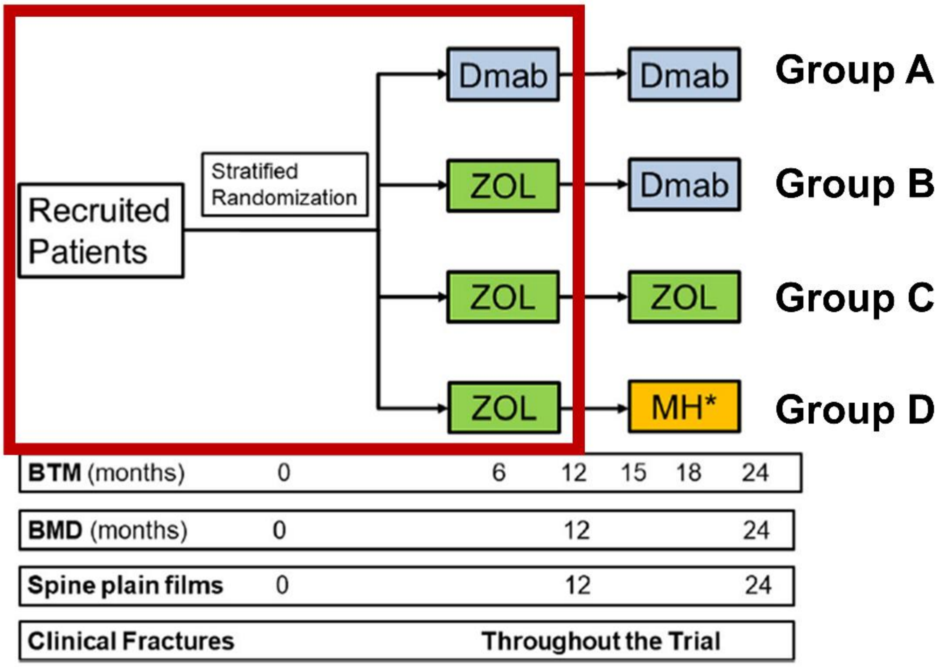

The red frame highlights the first year of the trial.

Abbreviations: Dmab, denosumab; ZOL, zoledronate; MH, monitored drug holiday; BTM, bone turnover markers; BMD, bone mineral density.

* An extra ZOL injection will be given once the C-terminal telopeptide levels raise above the pre-specified threshold, 0.573 ng/mL in post-menopausal women and 0.584 ng/mL in men, respectively.

© 2024 Lee CC et al. *JAMA Network Open.*

eTable 1. Results of Stratified Randomization

**Stratification by sex**	**Stratification by T-score**	**Group A**	**Group B**	**Group C**	**Group D**	**Case number in each stratum**
**Female**	T-score > -2.5	10	10	11	10	41
	T-score ≤ -2.5	13	13	14	14	54
**Male**	T-score > -2.5	1	1	0	1	3
	T-score ≤ -2.5	1	1	0	1	3
	Case number	25	25	25	26	Total number = 101

© 2024 Lee CC et al. *JAMA Network Open.*

eTable 2. Bone Mineral Density (BMD) Loss in the Lumbar Spine, Total Hip, and Femoral Neck Exceeding the Least Significant Change in the Zoledronate (ZOL) Group

Least significant change: Lumbar spine >5%, total hip>4%, femoral neck>4%

	**Age (median[IQR])**	**Weight (median[IQR])**	**BMD change (median[IQR])**
**ALL (n=73)**			
**LS(n=11)**	73.00[65.00, 75.50]	50.00[44.50, 53.35]	-7.35 [-9.99, -6.69]
**TH(n=6)**	67.00[63.75, 70.25]	51.25[44.13, 56.50]	-5.09 [-6.11, -4.31]
**FN(n=12)**	73.00[67.75, 78.00]	56.00[47.00, 58.50]	-5.17 [-6.93, -4.99]
**Denosumab use for ≤ 2.5 years (n=58)**			
**LS(n=5)**	71.00[67.00, 74.00]	53.00[52.00, 53.70]	-7.12 [-7.35, -6.99]
**TH(n=4)**	67.00[63.25, 68.75]	51.25[46.13, 59.25]	-5.99 [-6.28, -5.38]
**FN(n=9)**	72.00[68.00, 78.00]	55.00[47.00, 58.	-5.03 [-8.64, -4.98]
**Denosumab use for ≤ 3 years (n=61)**			
**LS(n=6)**	72.50[68.00, 77.75]	52.50[48.25, 53.53]	-7.06 [-7.29, -6.45]
**TH(n=4)**	67.00[63.25, 68.75]	51.25[46.13, 59.25]	-5.99 [-6.28, -5.38]
**FN(n=9)**	72.00[68.00, 78.00]	55.00[47.00, 58.00]	-5.03[-8.64, -4.98]
**Denosumab use for ≥ 3 years (n=15)**			
**LS(n=6)**	73.35[65.50, 76.25]	44.50[43.25, 48.75]	-8.67 [-10.45, -6.76]
**TH(n=2)**	68.50[65.75, 71.25]	50.00[46.50, 53.50]	-4.32 [-4.38, -4.26]
**FN(n=3)**	74.00[68.50, 76.00]	57.00[50.00, 65.50]	-5.31 [-5.84, -5.17]

LS: Lumbar spine; TH: Total hip; FN: Femoral neck

© 2024 Lee CC et al. *JAMA Network Open.*

eTable 3. Bone Mineral Density (BMD) Changes in Women Who Had Ever Suffered Vertebral Fractures in the Zoledronate (ZOL) Group (n = 63)

	**Median [IQR]**
**Age (years)**	71.00 [65.50, 75.50]
**Weight (kg)**	57.00 [49.00, 64.00]
**BMD change (%)**	
**LS**	-0.65 [-3.58, 2.68]
**TH**	0.48[-1.43, 2.39]
**FN**	0.81 [-2.53, 3.79]

LS: Lumbar spine; TH: Total hip; FN: Femoral neck

© 2024 Lee CC et al. *JAMA Network Open.*
