# Supplementary material for: Zoledronate Sequential Therapy After Denosumab Discontinuation to Prevent Bone Mineral Density Reduction: A Randomized Clinical Trial
Source: JAMA Netw Open. 2024 Nov 11;7(11):e2443899. doi: 10.1001/jamanetworkopen.2024.43899 (PMC11555552; doi:10.1001/jamanetworkopen.2024.43899)
Supplement: Supplement 3. — Data Sharing Statement [file jamanetwopen-e2443899-s003.pdf]

Data Sharing Statement

Lee. Zoledronate Sequential Therapy After Denosumab Discontinuation to Prevent Bone Mineral Density Reduction. *JAMA Netw Open*. Published November 11, 2024. doi:10.1001/jamanetworkopen.2024.43899

Data

Additional Information: Denosumab Sequential Therapy (DST), https://classic.clinicaltrials.gov/ct2/home, identifier: NCT03868033
Data available: No

Additional Information

Explanation for why data not available: The spreading of the data collected in this study are only available from the corresponding author upon reasonable request.
